# A systematic review of carbohydrate-based microneedles: current status and future prospects

**DOI:** 10.1007/s10856-021-06559-x

**Published:** 2021-07-31

**Authors:** Rupali S. Bhadale, Vaishali Y. Londhe

**Affiliations:** Shobhaben Pratapbhai Patel School of Pharmacy & Technology Management, SVKM’s NMIMS, Vile Parle [W], Mumbai, 400056 Maharashtra India

## Abstract

Microneedles (MNs) are minimally invasive tridimensional biomedical devices that bypass the skin barrier resulting in systemic and localized pharmacological effects. Historically, biomaterials such as carbohydrates, due to their physicochemical properties, have been used widely to fabricate MNs. Owing to their broad spectrum of functional groups, carbohydrates permit designing and engineering with tunable properties and functionalities. This has led the carbohydrate-based microarrays possessing the great potential to take a futuristic step in detecting, drug delivery, and retorting to biologicals. In this review, the crucial and extensive summary of carbohydrates such as hyaluronic acid, chitin, chitosan, chondroitin sulfate, cellulose, and starch has been discussed systematically, using PRISMA guidelines. It also discusses different approaches for drug delivery and the mechanical properties of biomaterial-based MNs, till date, progress has been achieved in clinical translation of carbohydrate-based MNs, and regulatory requirements for their commercialization. In conclusion, it describes a brief perspective on the future prospects of carbohydrate-based MNs referred to as the new class of topical drug delivery systems.

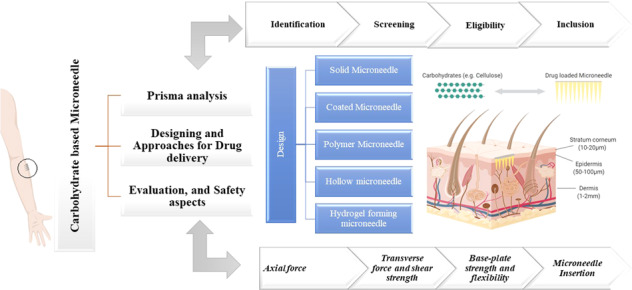

## Highlights

- The transdermal route can be a promising approach for drug delivery.
- Microneedles (MNs), a novel and important transdermal drug delivery system (TDDS), are discussed.
- Prisma guidelines are utilized to review carbohydrate-based MNs for medical applications.
- More innovative applications are predicted owing to their physiological features.

## Introduction

Over the last few decades, we have witnessed captivating technology development in drug delivery systems. This technology improvement has enhanced the therapeutic efficiency of biotherapeutics (e.g., vaccines and macromolecules) and hydrophilic drugs [1]. The therapeutic efficiency depends on the administration route, the aim is to overcome the challenges of the conventional drug delivery system [2]. The notable drawbacks of the oral and parenteral delivery system are the first-pass metabolism in oral dosage form and poor patient compliance, respectively. This led to the need to develop a delivery system with reduced dosing frequency, self administration, and better patient compliance, economical and eco-friendly [3, 4].

Emerging techniques, like the transdermal drug delivery system (TDDS), mainly focus on delivering therapeutic agents through the skin. Universally known, the transdermal route evades the first-pass metabolism, drug–food, and drug–drug interaction, thus enhancing the bioavailability of the therapeutic agents [5, 6]. An ideal candidate for TDDS is a hydrophobic drug molecule with potent action, low molecular weight (M/W ≤ 400 Dalton), and lower diffusion rate. Though an attractive alternative, TDDS possesses a major hurdle—stratum corneum, i.e., the skin barrier [3, 6].

**Fig. 1:**
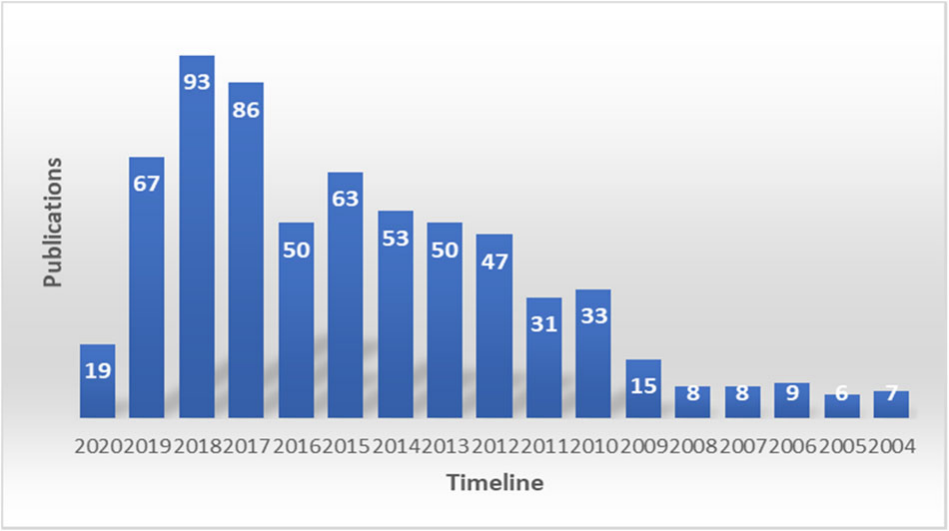
Cumulative publications on the microneedle drug delivery system. The analysis was done by the PubMed database using the search term “microneedle drug delivery” [98]

A three-dimensional, needle-like biomedical device that efficiently delivers the drug through the skin barrier without disturbing and overcoming the conventional method’s limitation, microneedles present themselves as a promising development for TDDS, comprising micron-size arrays organized in a small area [7, 8]. This device is developed with the aim to overcome the shortcomings of a hypodermic needle and transdermal patch. The first research was published in the late 2000s, and since then, the research activities have grown exponentially, as shown in Fig. 1.

A systematic review and meta-analyses (Prisma) are an evidence-based minimum set of records for disclosure of systematic review and meta-analysis. The preferred reporting records emphasize the propagation of studies by analyzing randomly assigned records and can be utilized as a critical element for disclosing systematic reviews of different research types, predominantly estimating the intrusion. These guidelines are designed to assist authors to enhance the presentation of systematic review and meta-analysis and used for journal peer reviewers and editors for critical evaluation of the published systematic review. It also delivers comprehensive procedures relating to the technique, including eligibility criteria, information source, search strategy to execute a systematic review, and meta-analysis [9].

In the past, a wide range of materials were used for the fabrication of MNs. Initially, MNs were fabricated using silicon [10–12], titanium oxide [13], stainless steel [14], ceramics [15], and glass [8]. Polymers play a vital role in the fabrication of MNs and a key pillar for clinical applications [16, 17]. Biopolymers, predominantly carbohydrates, were considered over synthetic polymers because of their biocompatibility, low toxicity, biometric characteristics, and biodegradable properties [18–20]. The parade of carbohydrates existing in nature has permitted the fabrication of MNs with exceptional features [18–23]. Several reviews on microneedles exist, but none exhaustively concentrated on carbohydrate-based MNs and reviewed using PRISMA guidelines [9]. Hence, considering the existing significance and the extent of carbohydrates, this review discussed challenges during the fabrication of carbohydrate-based MNs for drug delivery. Finally, it is highlighted with the prospect and clinical presence of carbohydrate-based MNs.

## Methods

### Eligibility criteria

A literature search was performed on English language papers using the PRISMA guideline [9]. Peer-reviewed articles published in the year January 2000–May 2020 were included, and publications before January 2000 were excluded. Studies describing carbohydrate-based MNs and studies related to the in vitro, in vivo data of carbohydrate-based MNs were chosen to be included. Articles published only in the English language were included.

### Search strategy

A search of the PubMed database identified studies published until May 2020. MEDLINE, Scopus, Google Scholar, Web of Science, and gray literature were incorporated into the search strategy. The search terms included ‘microneedle drug delivery’ and ‘carbohydrate-based microneedle’. It also included terms like clinical practices, bioavailability, fabrication, microneedle array, and patient compliance.

### Quality article and data analysis

The article quality and references of relevant articles were assessed individually according to the PRISMA protocol criteria. The quality criteria used to describe the studies were biopolymers, design, evaluation, and safety of the microneedle, preclinical, and clinical presence. A detailed description of the criteria used for the qualitative assessment of the records is given in Table 1.

**Table 1:** Description of criteria listed for the selection of records for review

Sr. No.	Criteria	Description
**1**.	Designing, evaluation, and safety of microneedle	• Was there a clear description of the fabrication, biopolymer used, and compatibility of carbohydrate-based microneedle? • Was there a clear description of the evaluation and application of carbohydrate-based microneedle?
**2**.	Preclinical and clinical presence	• Was a complete preclinical and clinical study described? • Was there a relevant conclusion to the study?

### Results of PRISMA analysis

**Fig. 2:**
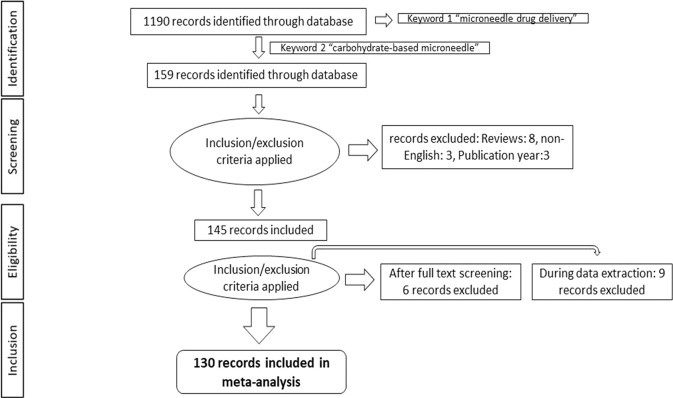
PRISMA flowchart for the records reviewed [9]

A literature search was conducted using PRISMA guidelines. Search strategies were set with different keywords and specific assessment criteria for inclusion and exclusion of records, as shown in Fig. 2. As of 1190 records identified from prime research-comprehensive databases, 159 papers were retained to include the keyword “carbohydrate-based microneedle” in the search strategy. Fourteen records were excluded as they were formed in other languages and review articles to give 145 inclusions. According to the inclusion criteria given in Table 1, from the 145 inclusions, 15 papers were further excluded during full-text screening and data extraction as they did not describe carbohydrate-based microneedles. Finally, a total of 130 records were considered for a systematic review.

## Results of Prisma analysis

### Basics of microneedles

#### Descriptions and features

**Fig. 3:**
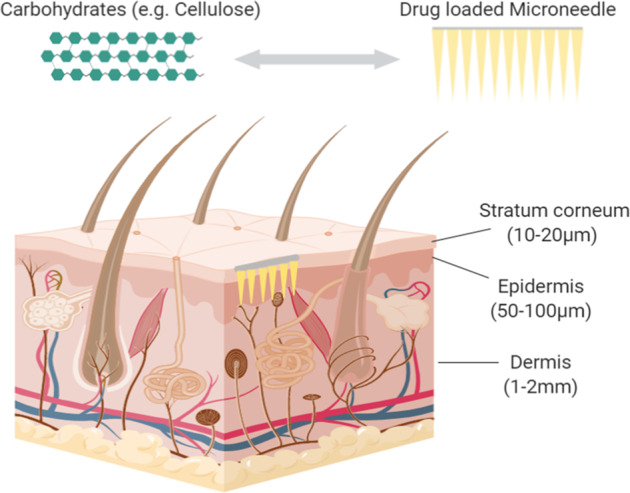
Schematic representation of a microneedle inserted into the skin

Microneedles are microscale arrays designed to penetrate the skin’s stratum corneum, forming microchannels through which active molecules diffuse passively (Fig. 3) [24–26]. Microneedles are 150–1500 µm in length, 50–250 µm in width, and 1–25-µm thick tips, enhancing the drug efficacy and transcutaneous flux of pharmaceuticals [27].

**Fig. 4:**
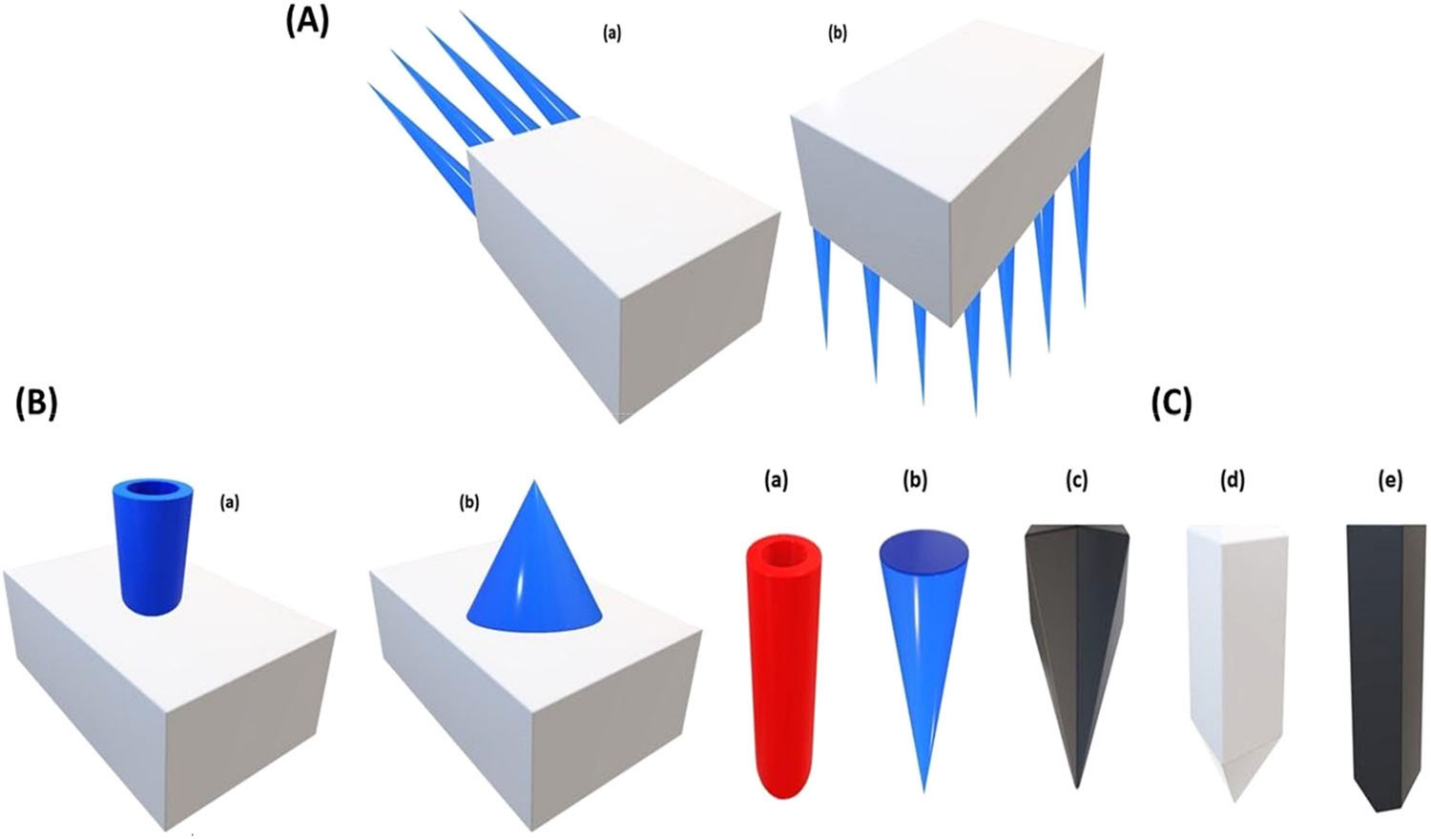
Schematic representation of MNs A MN assembly (**a**) in-plane and (**b**) out-of-plane. **B** The design of out-of-plane MNs is defined as (**a**) hollow and (**b**) solid. **C** Microarrays are (**a**) cylinder-shaped, (**b**) conical, (**c**) pyramidal, (**d**) and (**e**) pentagonal-based pointy and tapered tip, respectively

Generally, MNs are categorized based on their assembly (Fig. 4 (A)), design (Fig. 4 (B)), and tip shape (Fig. 4 (C)). MNs are manufactured into two elementary designs—in-plane and out-of-plane. The arrays of in-plane MNs (Fig. 4 (A) a) are parallel to the surface, whereas those of out-of-plane MNs (Fig. 4 (A) b) rise vertically from the base. The out-of-plane MNs can further be classified as hollow (Fig. 4 (B) a) or solid-shape MNs (Fig. 4 (B) b). Microneedle tips could be cylindrical (Fig. 4 (C) a), conical (Fig. 4 (C) b), pyramidal (Fig. 4 (C) c), and pentagonal with either a pointy or a tapered tip (Fig. 4 (C) d and e), accessible in many more forms [8, 28].

Microneedle arrays are optimized by choosing the array dimensions and design, which might inflict less pain, good mechanical strength, and drug delivery [28]. Gills H.S investigated that a decrease in microneedle length and microarrays reduces pain and establishes that there is no role of microneedletip angle, thickness, and width in the degree of pain caused by the application of MNs [29].

#### Approaches for drug delivery with MNs

**Fig. 5:**
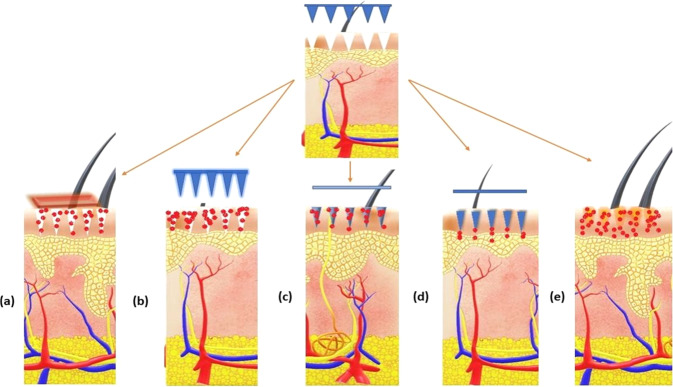
Different types of MNs according to the drug delivery approaches **a** solid, **b** coated, **c** dissolving, **d** hollow, and **e** hydrogel-forming MNs

The delivery of drug-using MNs is characterized according to five different types and related forms of drug delivery (Fig. 5, Table 2).

**Table 2:** Drug delivery approaches and fabrication methods for different types of microneedles with delivery efficacy

Systems	Approaches	Fabrication techniques	Delivery efficiency^a^			References
			Drug loading	Sustain release	Molecular range	
Solid MNs	Poke with patch	Vapor deposition, Dry etching, Wet etching, Micro-stereolithography, Laser cutting, laser ablation, Photolithography, Micro-molding, melt casting, and Metal electroplating, 3D printing	+	+	++	[34, 99]
Coated MNs	Coat and poke	Dipping, Spraying	+	+	++	[100]
Dissolving MNs	Poke and release	Photolithography, Deep X-ray lithography, drawing lithography, Micro-molding and melt casting, Droplet born air blowing, Two-photon polymerization, 3D printing	+++	+++	++	[27, 34, 101]
Hollow MNs	Poke and flow		+++	+++	++	[102, 103]
Hydrogel-forming MNs	Poke and release	Photolithography, Deep X-ray lithography, Dry etching, Wet etching, Metal electroplating, drawing lithography, Two-photon polymerization	+++	+++	+++	[104–106]

^a^Low- +, Moderate- ++, High- +++

##### Poke-and-patch approach

The first method is to poke the epidermis layer by microarray, which produces microchannels for drug delivery, subsequently detaching the microarray. Then, a transdermal patch is applied above it. Furthermore, to improve permeability, the electro field may be used. Solid microneedles follow this approach for the enhancement of skin permeability.

##### The coat-and-poke approach

The second way is to coat the microneedle arrays with the drug dispersion. The coated drug dissolves after piercing the coated microneedles into the skin. Microarrays can be covered with several compounds, including small molecules, proteins, plasmid DNA, virus particles, polymer nanoparticles, and water-soluble compounds.

##### The poke-and-release approach

As a novel alternative to coated microneedles, the drug is encapsulated within microneedles made up of polymers and polysaccharides for rapid and sustained release in the skin. As a result, after piercing into the skin, the microneedles dissolve or degrade, releasing the encapsulated drug in a controlled manner.

##### The poke-and-flow approach

In contrast to solid microneedles, hollow microneedles offer extra abilities and fewer difficulties. Hollow microneedles have complex fabrication processes with intrinsically weaker and sophisticated geometry. They permit the liquid formulations, which allow diffusion of the drug rapidly or with controlled flow rates, as required, but the rate-limiting step in hollow micro-arrays is the maintenance of flow rate.

#### Mechanical characterization of microneedles and safety evaluations

Mechanical characterization is a crucial step in the successful fabrication of MNs. Extensive stress during the insertion and removal of MNs is applied. Hence, it is essential to perform mechanical characterization and analyze the safety of MNs [11]. A variety of tests available to check mechanical strengths [30], are as given below.

##### Axial force mechanical test

**Fig. 6:**
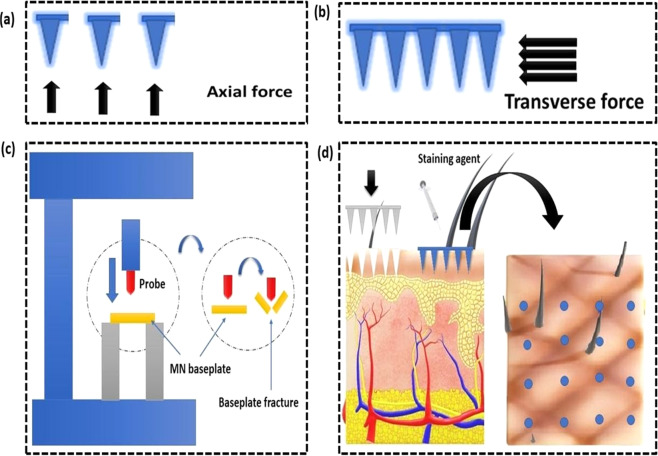
Illustration of the mechanical properties of MNs. **a** Axial force test. **b** Transverse force test. **c** Base-plate strength and flexibility tests. **d** Microneedle-insertion measurement by staining

In this test, force is applied to the MNs perpendicular to the base plate (Fig. 6a) [31]. The applied force can be analyzed using a mechanical station, which details both displacement and force against a hard surface at a definite rate [32, 33]. If a fracture occurs, a rapid drop can be seen in the force-displacement curve produced and considered as failure force [33]. A single microneedle (MN) is used to analyze failure force, which does not accurately simulate the pressures faced during insertion into the skin [30, 34, 35]. After piercing MNs into the skin, the forces are dispersed over a greater MN area as the flexible skin encases the MNs. The said axial force test is often used to assess polysaccharide MNs. Table 3, a summary of the published data about the mechanical strength of polysaccharide MNs [36].

**Table 3:** Failure force after an axial force load of carbohydrates

Composition of MN	Failure force	Reference
Hyaluronic acid (HA)	≥0.05	[107]
	0.4–0.6	[108]
	0.18	[109]
HA with Epidermal growth factor	0.63–0.78	[43]
Sodium hyaluronate with Enterovirus	≥0.08	[110]
Lysozyme loaded HA	0.20	[81]
Methacrylate HA	≥0.15	[48]
Sodium alginate	Transverse force failure 0.04	[32]
Chitosan	≥0.2	[82]
Magnetic graphene quantum dots loaded Chitosan	≥0.16	[111]
Carboxymethyl Cellulose	0.5–0.8	[108]

##### Transverse force and shear strength

Due to skin irregularities and elasticity, frequent incomplete insertion and bending of MNs arrays were observed. Thus, a transverse fracture force test is essential to investigate the performance of MN during application [32, 33]. The test is carried out on a mechanical test station; a transverse force (normal to the MN y-axis) is employed at a definite point on the MN array until the MN fractures [33]. Again, a rapid reduction in the force-displacement curve shows the MN defect [31]. This test is carried out on a row of MNs rather than a single array, which calculates transverse fracture force per MN (Fig. 6b). According to the literature, this test’s critical drawback is that the metal probe should be parallel with the MN array manually, which leads to inaccuracy [31, 32].

##### Base-plate strength and flexibility tests

Assessment of base-plate strength is crucial as the above-mentioned mechanical strength. Fracture of the base plates during piercing to the skin is not acceptable. Hence, base plates should be flexible enough to consent to the skin structure deprived of rupturing (Fig. 6c). A three-point bending test can be used to analyze base-plate strength. Donnelly et al. utilized a Texture analyzer to apply force with a metal probe to the base plate sitting between two aluminum blocks. Also, the base-plate flexibility is evaluated with the bending fracture of MNs [31].

##### Microneedle insertion measurement

Several techniques are applied to evaluate the insertion of MNs into the skin. Staining is a commonly used qualitative technique that stains epidermis cells, not microchannels created in the stratum corneum (Fig. 6d) [37–39]. Other qualitative methods are transepidermal water loss [39, 40] and electrical impedance [34, 35, 41] after poking the skin. Confocal microscopic imaging [36], histological cryosectioning with adjunct staining [41], and optical coherence tomography [31] was used, which estimates quantitative insertion-depth data. Lately, Larraneta et al. projected an artificial membrane for MN-insertion studies [42].

Factors like different materials and geometry of MN interrupt the insertion studies, using various experimental protocols and skin models. Thus, the standard protocol is crucial to assess mechanical performance.

##### Microneedles’ safety

Microneedles are based on the principle of piercing the outermost skin layer, which is not similar to the conventional transdermal patch. The suitability of microneedles has been evaluated and predicted on the degree of adverse effects, namely, itching, scaling, prickling, stinging, tightness, burning, edema, and erythema. No one has reported carbohydrate microneedle-related local infection. The disturbance may occur during the piercing or compatibility of the material used for fabrication with skin or residual solvents in the needles. The microneedle is designed to avoid pain while piercing. A formed microchannel must be reclosed in the short term to eradicate the contamination. The microneedles and formulation should be biocompatible with the skin. Modepalli et al. studied the safety and toxicity of hydrogel-forming microneedles on the human skin. They stated no toxicity in cell lines due to the microneedle [37]. Sodium hyaluronate and polyvinyl pyrrolidone used to fabricate dissolving microneedles were examined for cytotoxicity using an MTT assay to assess cell viability of more than 87% at the end of 72 h. The biological safety of ibuprofen sodium-loaded hydrogel-forming microneedles was studied and observed that no bioburden was traced [16]. Drug-loaded hyaluronic acid microneedles led to no allergic or irritant-contact dermatitis, hence, considered as a safe product for human use [43].

In certain cases, MNs pierce the epidermis and dermis layer, generally sterile areas of the body [7]. Thus, MNs must not contain microbes that may lead to systemic infections. Also, microbial penetration through MN-created holes is significantly low than conventional hypodermic needle puncture. The possible solution may be the production of sterile MNs, but the sterilization method should be carefully considered to evade alteration of key features of the product and increase production cost. For instance, aseptic manufacturing will be costly, terminal sterilization with moist heat or microwave heating, and gamma radiation could damage the MNs. McCrudden et al. evaluated the effect of different sterilization methods on dissolving and hydrogel-forming MNs [16]. Thus, safety studies can be performed for the polymeric matrix which will be used for the fabrication of MNs.

#### Carbohydrate-based microneedles

Carbohydrates play a quintessential role in the fabrication of MNs, and thus form the emphasis of this review. Carbohydrates, such as sugars, hyaluronic acid [44, 45], cellulose [46, 47], and chemically modified methacrylate hyaluronic acid [48], carboxymethylcellulose [49, 50], are utilized alone, in a blend, or as a composite. They are similar to the extracellular matrix and are simply recognized and accepted by the human body [51]. They are eliminated by metabolism and excretion through the renal system, evading tissue accretion [52]. Carbohydrates are approved by the US Food and Drug Administration (US FDA) and classified as generally recognized as safe (GRAS) [53, 54].

Carbohydrates reveal an enormous structural disparity and several intrinsic characteristics, which impact the mechanical properties and invasion ability of MNs and influence the biological potency and durability of the therapeutic agent [55–57]. Certain traits, like the polymer’s weight, swelling index, and an interface between the polymer and active agent, can stimulate and alter the drug-release rate [2, 58, 59]. The innumerable topographies impact their in vivo behavior and can be grouped into dissolvable (dextran, sodium chondroitin sulfate, and hyaluronic acid), biodegradable (chitin, starch), and swellable polymeric microarrays (methacrylate hyaluronic acid) as summarized in Table 4. The following section gives an overview of various carbohydrates. Table 5 highlights a few of the current examples of carbohydrate-based MNs described in the literature detailing their primary element, a therapeutic agent, and their biomedical applications.

**Table 4:** Merits and demerits of dissolvable, biodegradable, and swellable carbohydrate-based microneedles [54]

Microneedle design	Merits	Demerits
Dissolvable Microneedle	• Allow fast drug release as the rate of dissolution is increased • Encapsulation or coating can improve drug loading • Precise drug loading can be achieved • Generally used for sustained drug release	• A patch can not be detached before the complete dissolution of microneedles
Biodegradable Microneedle	• Encapsulation or coating can improve drug loading • Precise drug loading can be achieved • Attachment of Drug reservoir is allowed	• After few days of degradation, polymer residue can be detected in the skin
Hydrogel forming/Swellable Microneedle	• After removal, Smoothness prevents reinsertion • After few days of degradation, polymer residue can’t be detected in the skin • If necessary, treatment can be stopped	• Inadequate drug loading • Bioactivity of a drug may hamper due to cross-linking of the polymer

**Table 5:** Summary of carbohydrate-based MNs for drug delivery

Carbohydrate(s)and auxiliary agent	Active pharmaceutical ingredient (API)	Delivery efficiency	Findings	Reference
Maltose	Ascorbate-2-glycoside	Completely dissolves within 5 mins after application, in vivo	Short-term safety after insertion in healthy human skin and notable disposability	[62]
Maltose	Doxorubicin	Release the drug till 24 h, in vitro	Enhancement in permeation after piercing into human cadaver skin	[63]
Hyaluronic acid (HA)	All trans-retinoic acid, Tetanus toxoid, Ovalbumin, and diphtheria toxoid	Completely dissolves within 120 mins after application, in vivo	Better stability, induced immunological responses after 12month of storage	[109]
HA	Ovalbumin and adenovirus vector	Completely dissolves within 60 mins after application, in vivo	Improved response when compared with conventional vaccination	[64]
HA	Fluorescein isothiocyanate-dextran	Microarrays completely dissolve within 120 mins after piercing, in vivo and continuous dissolution of a baseplate seen after application of 7 h.	Enhanced permeability of high molecular macromolecule ass	[112]
HA	Insulin	Completely dissolves within 60 mins after application, in vivo	A dose-related hypoglycemic effect akin to a hypodermic needle	[74]
HA	Green tea extract	Release the drug till 72 h, in vitro	Diminution of microbial development (gram-negative and positive) at the infected area	[113]
HA	Enterovirus 71 virus-like particles	Completely dissolves within 2 mins after application, in vivo	API remained stable during fabrication, an increase in immunization by giving protection against hand-foot and mouth disease when compared with intramuscular injection	[110]
HA	Igg (Immunoglobulin G)	Dissolves within 10 mins after application, ex-vivo in human skin	Principal Protein was conserved, and its tertiary structure unchanged	[73]
HA with Amylopectin	Niacinamide and ascorbic acid	Dissolves within 8 h after application, ex-vivo in porcine skin	Applicable in cosmetic owing to its anti-oxidant activity	[114]
HA	Tuberculin purified derivatives	80% of dissolution /diffusion of MNs seen within 8 h.	Potential to be used for diagnosis of tuberculosis	[115]
HA	Adenosine	–	Demonstrated improvement or Similar outcomes compared to cream	[65]
HA with gold (Au) nanocages loaded	Doxorubicin	Dissolves within 5 mins after application, in vivo	Applicable for the devastation of superficial skin tumor	[69]
HA	Dermatophagoids farinae extract	Twice a week for 4weeks, extended-release formulation	Potential as allergen-specific immunotherapy	[116]
HA	Live attenuated Bacille Calmette-Guerin bacillus	Diffusion of the drug was observed in 6 h after application of MNs	Applicable for effective vaccine delivery when compared with the conventional dosage form	[66]
HA	Β-3-Adrenoceptor agonist and thyroid hormone	Completely dissolves within 2 mins after application, ex-vivo in porcine skin	The decrease in body fat and weight in obese mouse models	[107]
HA	Pegylated gold nanorod and doxorubicin	–	*The* in vitro study revealed that the photothermal effect destroyed completely A431 cells used for epidermoid cancer treatment	[117]
HA	Ascorbic acid 2-glucoside	Approximately, 75% of dissolution of MNs seen at the end of 20 mins.	The dose and activity of the Drug was sustained after e-beam sterilization	[70]
HA with Nanostructured lipid carriers using Compritol, labrafil	Nile red	Sustained release of Nile red	Effective delivery of hydrophobic component	[118]
HA and carboxymethyl cellulose + Amylopectin	Rhodamine B	Dissolves within 8 mins 10secs after application, ex-vivo in porcine skin	Enhanced permeability of Drug	[47]
HA and carboxymethyl cellulose + Amylopectin	Rhodamine B Niacinamide	Approximately, 40% of dissolution of MNs seen at the end of 10 mins.	Increased permeability, which can be applied for cosmetic	[47]
HA + 3-aminophenyl boronic acid-modified alginate	Insulin	Sustained release of Insulin achieved, in vivo	Initiation of a maintained hypoglycemic effect in diabetic mice	[119]
HA cross-linked with N, N-methylene bis (acrylamide) + nanoparticles of dextran	Anti-PD1 immunotherapy	A synergistic effect was observed, in vivo	Increased inhibition of tumor growth over the intratumoral injection	[67]
Sodium chondroitin sulfate + poly (vinyl pyrrolidone), lyotropic liquid crystal	Sinomenine hydrochloride	Sustained release of Sinomenine hydrochloride achieved to some extent, in vivo	Enhanced permeability of Drug with the maintained release, which can be applied to adjuvant arthritis model rats	[120]
Sodium chondroitin sulfate	Capsaicin	Completely dissolves within 20 mins after application, in vitro	Improved efficiency over a topical formulation which exhibits rapid local analgesic action	[121]
Sodium chondroitin sulfate	Insulin	fully-loaded tip and partially loaded tip with Insulin exhibit similar dissolution rate	Better efficiency of the Drug, fully-loaded tip exhibit extreme effect of the Drug detected at 1.7 ± 0.2 h and 1.5 ± 0.2 h for the partially loaded tip	[122]
Carboxymethyl cellulose	Recombinant human adenovirus type 5 vector encoding HIV-1 gag	Rapid dissolution was seen, in vivo	Long-lived antigen-specific cell genesis on the mucosal surface to employ local immunosurveillance, which supplies the first-line defense against pathogens	[80]
Carboxymethyl cellulose	Valproic acid	At the end of 90 mins, drug release enhanced due to dissolution of MNs	Enhances hair growth with high precision over the topical system	[81]
Carboxymethyl cellulose	(Anti-TNF-alpha-Ab)-HA conjugates	Approximately, 60% of dissolution of MNs seen at the end of 20 mins after application	Applicable to a wide array of inflammatory skin diseases	[123]
Carboxymethyl cellulose + Amylopectin	Rhodamine Ascorbic acid	Approximately, 30% of dissolution of MNs seen at the end of 8 mins after application, ex-vivo in porcine skin	3-fold permeability enhancement of rhodamine and 6-fold rise anti-oxidant activity of ascorbic acid over the topical application	[49]
Carboxymethyl cellulose + double hydroxides nanoparticles	Ovalbumin	Completely dissolves within 1 mins after application in skin	Efficacy enhancement was perceived over the subcutaneous injection	[106]
Carboxymethyl cellulose	*Lactobacillus*	Immediately dissolved after application, in vivo	The in vivo study revealed the maximum concentration of lactic acid in pig and rat skin	[124]
Carboxymethyl cellulose + hydroxymethyl cellulose	Donepezil hydrochloride	Approximately, 80% of dissolution of MNs seen at the end of 60 mins after application, ex-vivo in porcine skin	Enhancement in response over the oral route for Alzheimer disease	[79]
Carboxymethyl cellulose + poly (vinyl alcohol) as a backing membrane	Etonogestrel microcrystals	Approximately, 70% of MNs dissolved at the end of 60 mins after application, in vivo	Tip loaded MNs does not affect mechanical properties and more consistent plasma level over the intradermal injection	[125]
Carboxymethyl cellulose + poly (methyl vinyl ether co-maleic anhydride)	Lidocaine hydrochloride	Completely dissolves within 5 mins after application in rat skin	A stable formulation for 3months and higher dissolution over the commercial cream but less efficiency	[126]
Chitin	Purified protein derivative, containing a mixture of antigen	–	The definite results approved the potential application of Chitin MNs for diagnosis	[83]
Chitosan+ Trehalose, poly (vinyl alcohol)/ poly (vinyl pyrrolidone)	Luteinizing hormone-releasing hormone analogs, goserelin	Sustained release of drug for 28days	Significantly applied for the treatment of androgen-deprivation, castration level was sustained for 14days	[127]
Chitosan+ β-Sodium glycerophosphate and hydroxypropyl β-cyclodextrin	Levonorgestrel	Approximately, 57% of MNs dissolved at the end of 120 mins after application	Alike pharmacokinetic parameters over the oral dose with steady plasma concentration	[128]
Chitosan+ Poly (vinyl alcohol) + Polyvinylpyrrolidone as supporting array	Ovalbumin	Controlled release of drug	Increased efficiency of the low-dose Drug when compared with intramuscular injection	[94]
Chitosan+ Magnetic graphene quantum dots + Polyethylene glycol	Lidocaine hydrochloride	Microarrays dissolved within 5 mins after application	Enhanced release from 25.7 to 96.4% due to iontophoresis	[111]
Starch+ Gelatine	Bovine serum albumin	Sustained release	Sustained action for 8days	[82]
Starch	Insulin	Immediately dissolved after application	Substantial pharmacological action distributed the whole Drug within 5 mins	[89]
Hydroxyethyl starch (tip)+ Sodium chondroitin sulfate (needle)	Hepatitis B surface antigen (tip)	100% dissolution was achieved after application	Similar immunoreaction as a marketed vaccine, antigenicity was reserved at 37 and 45 °C	[129]
Pullulan	FITC-BSA and insulin	Completely dissolves within 10 mins after application in skin	The efficiency of drug administration using MNs was more effective than the oral route; these MNs could replace the current method for protein/peptide delivery	[90]
Dextran	Poly-L-arginine	Approximately, 75% of MNs dissolved after application in rat skin	Efficacy of Drug depends upon dosage regimen, also can be a substitute to skin allergy device	[130]

**Table 6:** list of active and completed clinical trials [91]

Carbohydrate	Condition	Identifier	Titles	Year	Status
HA	Crow’s Feet Wrinkles	NCT02497846	TEOSYAL^®^ PureSense Redensity [I] Injection Using MicronJet^®^ Needle in the Treatment of Crow’s Feet Wrinkles	2016	Completed
HA	Psoriasis	NCT02955576	Microneedle Patch for Psoriatic Plaques	2017	Active
HA	Vitiligo - Macular Depigmentation	NCT02660320	Comparison of the Efficacy of Micro-holes vs. Laser-assisted Dermabrasion for Repigmenting in Vitiligo Skin (Dermabrasion)	2018	Active
Starch	Primary Axillary Hyperhidrosis	NCT03054480	Fractional Micro-Needle Radiofrequency and Botulinum Toxin A for Primary Axillary Hyperhidrosis	2017	Completed
Starch	Primary Axillary Hyperhidrosis	NCT02823340	Fractionated Microneedle Radiofrequency for Treatment of Primary Axillary Hyperhidrosis	2016	Active

##### Sugars

Sugars present an attractive resource for the fabrication of biomolecules delivering MNs. Also, the combination with other polymers may enhance the mechanical strength and stability of MNs. Maltose is the most widely used sugar for the fabrication of MNs and is classified as GRAS [60]. Additionally, mannitol, trehalose, sucrose, xylitol, and galactose also can be utilized to fabricate MNs [61]. Some studies on sugar MNs are carried out by Miyano et al., who designed ascorbate-2-glycoside-loaded sugar MNs that depicted short-term safety in healthy human skin [62], while Nguyen et al. fabricated doxorubicin-loaded maltose MNs that showed the enhancement in permeation after piercing into human cadaver skin [63].

##### Hyaluronic acid (HA)

Hyaluronic acid is a crucial element of the extracellular matrix and cartilage, which has mucoadhesive properties [2, 54]. HA is a negatively charged biopolymer, and its salt has a high solubility in water. Matsuo et al. fabricated dissolvable HA MNs of different sizes that dissolved within 5 mins [64]. Kang et al. [65] prepared adenosine-loaded hyaluronic acid MNs for cosmetic purposes and observed that needles were completely dissolved within 15 mins after inserting into the porcine skin, which showed better results than when compared with the topical cream. Rather than adding the therapeutical element into the matrix, Liu et al. [44] fabricated MNs with tips loaded with hyaluronic acid dissolved within the 30 s. In this study, topical delivery of exendin-4 was studied, and permeability was carried out by labeling with fluorescein isothiocyanate (FITC). The results of in vivo studies depict burst release within the first 30 s, and almost-labeled dextran was dissolved within 5 min. MNs generated equivalent pharmacokinetic profiles when compared with subcutaneous injection in type-2 diabetic rats. Thus, it can be a substitute for type-2 diabetes therapy.

In a separate study, Chen et al. [66] fabricated *Calmette–Guerin* bacillus-loaded HA MNs, for tuberculosis prevention, which dissolved within 10–15 mins. As stated by the World Health Organization (WHO), the *Calmette–Guerin* bacillus vaccine is stable at 2–8 °C. This research reported good stability at ambient temperature and quick onset of action for the undeveloped area. For nanomedicine delivery, Wang et al. [67] formulated pH-sensitive dextran nanoparticles loaded in cross-linked HA MNs to treat skin cancer. The nanoparticles comprised dextran derivative, alginate, glucose oxidase, and aPD1 in an acid-degradable polymeric matrix. When applied to the melanoma site, this microdevice converts glucose to gluconic acid, producing an acidic environment and subsequent antibody release.

Another study involved direct incorporation of cells and polymer, like the adipose-derived stromal vascular fraction cells carrying HA MNs, for diabetic wound healing. Cells loaded with HA MNs depicted improved wound healing compared with HA MNs and isolated sections, proposing an alternative approach [68]. In a different light, Park et al. designed amylopectin MNs to deliver essential nutrients, specifically niacin and ascorbic acid, wherein the amylopectin was used to enhance the mechanical strength of HA MNs in a ratio of 1:2.3 (HA:amylopectin). The higher concentration of amylopectin resulted in a stiffer and brittle structure, tough to remove from the mold without breaking. Dong et al. [69] formulated Au nanocages and doxorubicin-loaded HA MNs as Au nanocages reinforce the mechanical endurance and photothermal effect of a drug on exposure to infrared light to results in the eradication of exterior skin tumors.

In another approach to customizing drug delivery, Kim et al. [70] designed HA MNs with trehalose and polyvinylpyrrolidone to facilitate peptide delivery. The study implied that polyvinylpyrrolidone prevents peptide agglomeration and enzymatic degradation, thus enhancing the dissemination of the drug into the systemic circulation. MNs for disease diagnosis were also performed. Chang et al. [48] developed swellable MNs with methacrylate HA for metabolic detection, wherein the MN patch extracts sufficient interstitial fluid in a shorter time without the need of any external device.

Cross-linking of HA may cause swelling and tailor the degradation or drug delivery [59, 71]. Zhang et al. [59] designed cross-linked HA MNs with 1,4-butanediol diglycidyl ether, enhancing the mechanical strength of MNs and lowering degradation of the matrix, thus, an excellent prospect for prolonged drug delivery. Larraneta et al. defined an additional cross-link, Gantrez S97, who developed hydrogels with HA and a copolymer of methyl vinyl ether and maleic acid, respectively. Gantrez concluded that esterified cross-linked HA MNs might release up to 48 h, which eased microbial infection [72].

The incorporation of proteins and peptides was also studied. Monkare et al. developed HA MNs loaded with IgG, which recovered 82% of the protein with no change in the tertiary structure after fabrication [73]. Liu et al. studied the stability of fabricated insulin-loaded MNs at different temperatures for a month and reported MN stability, with more than 90% insulin detection. Insulin release from such MNs was enhanced after storage and resulted in a glycemic-level reduction from 43 to 88% [74].

In different prospects, HA’s feasibility was demonstrated by e-beam sterilization, which impeded the ascorbic acid 2-glucoside degradation of HA MNs as the dissolution rate and drug release, structural features, and fracture force remained unaffected, without activity loss of capsulated active elements [70].

##### Chondroitin sulfate MNs

Sodium chondroitin sulfate is a hydrophilic component [75] of the extracellular matrix and cartilage [2, 59], which is immeasurably used for fabrication. Fukushima et al. fabricated human growth hormone, desmopressin-loaded sodium chondroitin sulfate, and dextran MNs, which established that dose and concentration were directly proportional to each other [57]. Ito et al. developed leuprolide acetate-loaded sodium chondroitin sulfate MNs that delivered drugs within 15 mins when applied to rat skin [76].

##### Cellulose-based MNs

Hydrophilic polymers like carboxymethyl cellulose and (hydroxypropyl) methylcellulose are predominantly utilized because of their several features. These biopolymers are used as thickening agents, binders, stabilizers, and film-forming agents [77, 78]. Kim et al. fabricated donepezil hydrochloride array tip-loaded (hydroxypropyl) methylcellulose MNs to treat Alzheimer’s disease. In vivo studies revealed that the release of the drug was achieved within 5 mins after insertion, and microarrays were completely dissolved within 15 mins in the skin [79].

On the other hand, Zaric et al. [80] fabricated human adenovirus type-5 vector-loaded carboxymethyl cellulose and sucrose (protein stabilizer) MNs for vaccine delivery. This research established the potential of MNs to sustain the antigenicity of attenuated vaccines after freeze-drying. Another study was performed on Lactobacillus-loaded carboxymethyl cellulose MNs, enhancing local skin health and rat’s immune functions [80], while Lahiji et al. fabricated valproic acid-loaded carboxymethyl cellulose MNs to treat hair regrowth. Enhancement in the valproic acid release was observed and compared with the topical formulation, which elevates hair follicles’ regrowth [81].

##### Chitin and chitosan MNs

Chitin and chitosan, after cellulose derivatives, are the widely used biopolymers produced by the deacetylation process. Chitosan comprises a high-density positive charge due to its acidic nature, allowing tissue and mucoadhesion [82–84]. Degradation of chitosan occurs by hydrolysis of acetylated residues, and its degradation rate is related to molecular mass and the degree of deacetylation [52]. Apart from these, the FDA has identified chitosan as GRAS, used for wound dressing and cartilage repair [54, 85, 86], and utilized for the fabrication of MNs for controlled and sustained drug delivery [85].

Jin et al. formulated chitin MNs coated with antigens, a convenient diagnostic tool for tuberculosis. Chitin MNs were water-insoluble, mechanically robust, physiologically inert, and exhibited a lower swelling index. Chen et al. [83] prepared chitosan MNs to allow the sustained release of bovine serum albumin (BSA). These chitosan MNs when pierced into porcine skin showed a 20% burst release of BSA after 30 mins. Subsequently, 95% of BSA was released within eight days. Within this period, chitosan MNs soften and break after insertion, which results in the retention of some polymeric pieces in the skin [83]. Besides this, Chen et al. [82] also developed tip-loaded chitosan MNs comprising model antigen ovalbumin for sustained delivery. These effective chitosan MNs contained biodegradable baking of poly (L-lactide-co-D, L-lactide), overcoming impression after piercing, which is not detected post 6 h. The in vivo study revealed that 50% of the antigen was released within the first two days, and a slight fluorescence was detected in three weeks [82].

##### Starch-based MNs

Starch is a biodegradable polymer extensively used due to its ease of processing and filmogenic ability for biomedical purposes [87]. While formulating, owing to its brittle behavior, starch is combined with other polymers [88]. Ling et al. substantially mixed starch with gelatine to transmit insulin, which loaded within 5 mins. Captivatingly, more than 90% of insulin was stable at ambient temperature for 30 days, specifying cost-effectiveness and convenience after application [89].

Pullulan (PL), a hydrophilic biopolymer formed by the yeast-like fungus *Aureobasidium pullulans* in starch and sugar cultures, plays a crucial role in biomedical applications. This biopolymer has unique characteristics, such as oxygen-barrier property, mechanical robustness, chemically inert, transparent, and filmogenic ability with no toxicity or mutagenicity. Hence, PL is an outstanding candidate for the fabrication of dissolvable MNs. Vora et al. fabricated PL MNs to deliver proteins and peptides, namely FITC-BSA and insulin. Ex vivo studies demonstrate protein/peptides detected within 15 mins when applied to porcine skin [90].

#### Clinical presence and regulatory requirement for commercialization

Many nonclinical studies were conducted for carbohydrate-based microneedles, which revealed enhancement in efficiency and permeability, but only a few accomplished positive clinical testing results. Table 6 summarizes active and completed clinical trials using carbohydrate-based microneedles [91]. Since microneedles are novel drug delivery systems, there are no particular requirements established to date. The conventional transdermal patch is simply applied to the skin’s exterior surface, whereas MNs pierce the stratum corneum and even interrupt the epidermis and dermis. This mechanism motivates the necessity of regulatory demands. Henceforth, new provisions should be defined for the MN, apart from the existing transdermal patch. Some essential regulations that need to be considered with the commercialization of MN arrays are given below:

1. Materials, length, sharpness, needle characteristics, and geometry should be correctly designed
2. The MN arrays should retain satisfactory microbiological standards
3. The MN arrays should keep the uniformity of content
4. Quality manufacturing, safety, and packaging should be maintained
5. Safe and nonhazardous procedures should be drawn for MN arrays
6. A self-disabling mechanism assuring a single use may require the MN arrays to avoid reuse by patients or others.
7. If MN arrays are reusable, cleaning information should be provided
8. For long-term use, material safety should be considered with precision
9. Labeling should contain instructions for use
10. MN arrays should be used with a proper applicator for proper insertion and pain-free delivery. Also, arrays should give reproducible results without complications
11. Immunological safety assurance should be considered

## Conclusion and future perspective

Drug delivery via the transdermal route has seen incredible development, among which the microneedle arrays facilitating drug infusion via forming several microchannels in the stratum corneum have grown exponentially over the past two decades. In the past fifteen years, the aspect of material science has transformed radically, and the utilization of natural polymers for recuperative purposes would be a critical success [53, 92, 93].

Carbohydrate usage presents an inclusive array of benefits owing to their intrinsic physiological compatibility, degradability, nontoxic, and sustainable nature. The inherent variation in carbohydrates, stemming primarily due to animal and plant origin, absence of standardization, and variation in appearance, with time or development, are few aspects that play a significant drawback role during commercialization [92]. The appropriate assessment and complete description of carbohydrates are necessary for analysis to overcome these drawbacks. Additionally, a significant feature emphasizes the probability of degeneration, decay, or adulteration of these resources during mining and development. The mechanical properties of carbohydrates can be enhanced by integrating with additional biopolymers [89], or nanosystem [69], and also cross-linking [94], which allows rupture of skin. The batch-to-batch variation impacts on mechanical qualities of a biopolymer.

Restructuring the study protocols as per the properties of the carbohydrates is essential. For instance, in moisture-absorbing polymers, assessments forecasting storage in water-resistant packages should be conducted. Along the same lines, considering the probabilities of utilizing these microarrays in states with high RH like the WHO climatic zone IV, deficiency of availability, and setups, appropriate contingencies should be planned [89]. The critical feature of MNs is the ease of self-administration. For bulk production and self-utilization, a unique applicator should be accompanied to allow adequate application, resulting in increased costs, but it is essential to ensure a preset force to assure skin insertion and its consequent advantages. Instability of some drug molecules to the fabrication step necessitates stabilizers into the polymeric formulation, thus stabilizing the drug during fabrication and long-term storage.

The actual application of MNs delayed as the regulatory guidelines are ongoing. Still, the academy is bringing novel instigating records in this zone, which highlights the difficulties during developments. The formation of policies to manufacture and analyze MNs is authoritative to ensure specifications and precision during production. Larraneta et al. [95] explained the need for specification for the analysis of MNs. The small MN tips are safe and minimally invasive. The critical principle of MNs is piercing the skin by generating microchannels, which reconcile after a few hours and release the active medicament [96]. However, the skin’s microchannels may allow bacteria entry, though the probability of bacterial contamination is significantly lesser than conventional injection [97]. The incidence of infection can be reduced by applying an alcoholic solution before applying MNs [96]. Piercing of MNs provides a pro-inflammatory microenvironment and immunological effects when applied frequently. Thus, the efficacy of MNs can be regulated by the skin’s mechanism. Currently, applicators are used to pierce the MN patch in the skin with a defined force. Variability can be seen when applied by a different candidate. The responsive biomarkers are used to assure that MNs are effectively applied.

Sterilization of the MNs is a crucial step that impacts the stability of the active pharmaceutical agent. Concerning the storage condition, drug-loaded MNs must be stable at ambient temperature for their intact mechanical properties and pharmacological performance. Another critical aspect emphasizes MN arrays’ disposal, which may transfer blood-borne disease through blood or ISF [97]. Nanocarriers or nanostructured polymer loaded carbohydrate MNs are widely used for drug delivery and diagnosis. This technique can be a substitute for the conventional dosage. Nowadays, MNs can detect pH or change in temperature.

In conclusion, sophisticated design and impressive fusion of carbohydrate-based MNs effectively deliver several active pharmaceutical agents with a broad spectrum of applications in various zones. Carbohydrate-based MNs have a fundamental part in wellness management, which provides stimulating revolutions in drug delivery. In these arrays, it is anticipated that such designs might be legalized in clinical practice, fetching motivation from nature into day-to-day drug delivery applications in a couple of years.
